# *Escherichia coli* ‐associated follicular cystitis in dogs: Clinical and pathologic characterization

**DOI:** 10.1111/jvim.16719

**Published:** 2023-05-08

**Authors:** Sanna J. Viitanen, Laura Tuomisto, Nina Salonen, Katariina Eskola, Kristel Kegler

**Affiliations:** ^1^ Department of Equine and Small Animal Medicine, Faculty of Veterinary Medicine University of Helsinki Helsinki Finland; ^2^ Department of Veterinary Biosciences, Pathology and Parasitology Unit, Faculty of Veterinary Medicine University of Helsinki Helsinki Finland

**Keywords:** canine, histopathology, in situ hybridization, urinary

## Abstract

**Background:**

Follicular cystitis is an uncommon inflammatory change in the urinary bladder wall characterized by the formation of tertiary lymphoid structures (TLSs) in the submucosa.

**Objectives:**

To characterize clinical and pathologic features of follicular cystitis in dogs and to explore in situ distribution and possible role of *Escherichia coli* as an associated cause.

**Animals:**

Eight dogs diagnosed with follicular cystitis and 2 control dogs.

**Methods:**

Retrospective descriptive study. Dogs diagnosed with follicular cystitis (macroscopic follicular lesions in the urinary bladder mucosa and histopathologic detection of TLSs in bladder wall biopsies) were identified from medical records. Paraffin embedded bladder wall biopsies were subject to in situ hybridization for *E. coli* 16SrRNA identification.

**Results:**

Follicular cystitis was diagnosed in large breed (median weight 24.9 kg, interquartile range [IQR] 18.8‐35.4 kg) female dogs with a history of chronic recurrent urinary tract infections (UTIs; median duration of clinical signs 7 months, IQR 3‐17 months; median number of previous UTIs 5, IQR 4‐6). Positive *E. coli* 16SrRNA signal was detected within developing, immature and mature TLSs in 7/8 dogs, through submucosal stroma in 8/8 dogs and within the urothelium in 3/8 dogs.

**Conclusions and Clinical Importance:**

Chronic inflammation associated with an intramural *E. coli* infection in the urinary bladder wall represents a possible triggering factor for the development of follicular cystitis.

**Abbreviations**
FDCs: follicular dendritic cells
IBC: intracellular bacterial community
IHC: immunohistochemistry
IQR: interquartile range
ISH: in situ hybridization
QIR: quiescent intracellular reservoirs
TLSs: tertiary lymphoid structures
UPEC: uropathogenic *E. coli*
UTIs: urinary tract infections

## 1 INTRODUCTION

Follicular cystitis, also known as *cystitis follicularis*, refers to a nonspecific inflammatory change in the urinary bladder wall characterized by the formation of tertiary lymphoid structures (TLSs) in the submucosa.^1^, ^2^, ^3^ The condition is well‐recognized but incompletely understood in humans and has been sporadically described in dogs, cattle, and buffaloes.^1^, ^4^, ^5^ Current evidence suggests that formation of TLSs in the urinary bladder occurs secondary to chronic inflammation in response to infection, bladder neoplasia, or chemical irritants allowing a highly localized immune response.^6^, ^7^, ^8^, ^9^ TLSs are transient and can disappear after elimination of the antigenic stimulation.^3^, ^6^

Dogs and humans share the commonality of high incidence of UTIs often resulting in recurrent or chronic cystitis.^10^, ^11^ From all, *Escherichia coli* is a major causative agent.^11^, ^12^ *E. coli* strains cultured from UTIs, referred to as uropathogenic *E. coli* (UPEC), cluster within the extraintestinal pathogenic *E. coli* group.^13^ Chronic and recurrent bacterial urinary tract infections (UTIs) are described to be associated with follicular cystitis in humans.^6^, ^7^, ^14^ Even though follicular cystitis has been occasionally described in connection with persistent bacteriuria and recurrent UTIs also in dogs,^4^, ^15^, ^16^, ^17^ the implication of *E. coli* in the pathogenesis of follicular cystitis and its possible repercussion in diagnosis and treatment has not yet been explored in samples from clinically ill individuals with natural UTIs.

Therefore, the aim of this study was to investigate and characterize in detail the clinical and pathologic features of follicular cystitis in dogs, as well as to explore the in situ distribution and possible role of *E. coli* as an associated cause.

## 2 MATERIALS AND METHODS

### 2.1 Study population

Medical records at the Veterinary Teaching Hospital of the University of Helsinki were retrospectively reviewed between January 2015 and February 2022 and dogs diagnosed with follicular cystitis were included in the study. The diagnosis of follicular cystitis was based on typical macroscopic appearance of follicular lesions in the urinary bladder mucosa and histopathologic detection of lymphoid follicles in bladder wall biopsies. Dogs with incomplete diagnostic work‐up or lacking adequate bladder wall biopsies were excluded.

Control biopsies from urinary bladder wall were obtained from dogs euthanized at the Veterinary Teaching Hospital of the University of Helsinki and donated for research and teaching purposes. Control dogs did not have signs of urinary tract disease or any known urinary tract disease before euthanasia and did not have bacterial growth in urine culture.

### 2.2 Diagnostic testing, sample collection

All dogs with follicular cystitis underwent a full clinical examination and blood samples for hematology and serum biochemistry were obtained from cephalic or jugular vein. Urine samples for urinalysis and bacterial culture were obtained either by cystocenthesis or during cystoscopy via aspiration through the working channel of the endoscope.

Abdominal ultrasound examination (EPIQ 7G, Koninklijke Philips N.V., Eindhoven, Netherlands) with a focus on the urinary tract was performed by an experienced radiologist. Intravenous urography (Iomeron 350 mg/mL injection solution, Bracco Imaging SpA, Milan, Italy) using computed tomography imaging (GE LightSpeed VCT 64, GE Healthcare, Fairfield, Connecticut) was performed in selected cases to visualize ureteral anatomy. Vaginoscopy and cystoscopy were performed under general anesthesia using sterile a 4.4 mm flexible (GIF‐N180, Olympus Europa SE&Co. KG, Hamburg, Germany) or a rigid 3.8 mm (Karl Storz SE & Co. KG, Tuttlingen, Germany) endoscope and images were acquired and stored for later analysis. Biopsies were acquired using sterile single‐use endoscopic forceps (EndoJaw FB‐231D.A, Olympus Europa SE&Co. KG, Hamburg, Germany) through the working channel of the endoscope and placed either in 10% neutral‐buffered formalin for histopathology or into a transport tube (Transystem M40 Amies Agar Gel Transport Swab, Copan Diagnostics, Murrieta, California) for bacterial culture. Biopsies were fixed in 10% neutral‐buffered formalin for no longer than 72 hours before routine histologic procedure.

In control dogs, urinary samples were obtained via cystocenthesis and full thickness bladder wall biopsies were obtained via laparotomy immediately after euthanasia.

### 2.3 Sample handling and laboratory analysis

Hematology (Advia 1800, Siemens AG, Erlangen, Germany) and serum biochemistry (Konelab 30i Clinical Chemistry Analyzer, Thermo Scientific, Fischer Scientific Oy, Vantaa, Finland) as well as urine analysis using conventional laboratory methods were performed without delay after sampling.

For microbiology, a 10 μL volume of urine specimens was quantitatively cultured onto tryptone soya agar with 5% sheep blood (TSA‐SB, Oxoid Ltd., UK). The urine culture plates were incubated at 35 ± 2°C aerobic atmosphere with 5% CO_2_ addition, and were evaluated at 24 and 48 hours. The biopsy specimens were cultured both aerobically (TSA‐SB and Chocolate Agar, Oxoid Ltd., UK) and anaerobically (Fastidious Anaerobe Agar with horse blood, F.A.A., Oxoid Ltd., UK), and additionally enriched in fastidious anaerobe broth (FAB, Tammer BioLab, Finland). The plates and the enrichment broth tubes were incubated at 35 ± 2°C and were evaluated at 24 hour intervals for 7 consecutive days. Until January 2016, the bacterial isolates were identified using conventional biochemical methods.^18^ Matrix‐assisted light desorption/ionization‐time‐of‐flight (MALDI‐TOF) mass spectrometry (Bruker Maldi Biotyper Microflex LT, Bruker Daltonik GmBH, Germany) was used for species identification from January 2016 onwards. The amount of bacterial growth was reported in colony forming units per ml for urine specimens, and using a semi‐quantitative scale (no growth/scarce/+/++/+++) for biopsy specimens. The antimicrobial susceptibility testing was performed according to the Clinical & Laboratory Standards Institute (CLSI) guidelines.^19^, ^20^

### 2.4 Histopathology, immunohistochemistry, and in situ hybridization

Paraffin embedded bladder wall biopsies obtained from dogs diagnosed with follicular cystitis were sectioned at 4 μm thickness and stained with hematoxylin and eosin (HE) or further processed for immunohistochemistry (IHC) and in situ hybridization (ISH).

Characterization of TLSs was performed on HE sections as previously described by Gulinac et al., 2020^9^ according to the degree of development at the time of biopsy sampling as follow: mild mononuclear infiltrate (up to 200 cells) with partial nodular arrangement (type 1 TLSs, developing); more pronounced mononuclear cell infiltration with more than 200 cells organized in well‐defined nodules without germinal center (type 2 TLSs, immature); and mononuclear cell infiltration in nodular aggregations with well‐defined germinal centers (type 3 TLSs, mature/terminal).

For IHC, antibodies used to phenotype inflammatory cells within the submucosa and TLSs were CD3 (rabbit polyclonal, A0452, Dako, Santa Clara, California) for T‐lymphocytes, CD79a (mouse monoclonal, HM57, Bio‐Rad AbD Serotec, Kidlington, UK) for B‐lymphocytes, and Iba‐1 (rabbit polyclonal, O19‐19741, FUJIFILM Wako Pure Chem. Corp., Osaka, Japan) for macrophages/follicular dendritic cells (FDCs).

In situ hybridization for bacterial RNA detection was performed with RNAscope technology (Advanced Cell Diagnostics ACD bio, Newark, California) using RNAscope 2.5 HD Reagent Kit‐Red (cat. 322350, ACD bio) and a probe specifically targeting *E. coli* 16SrRNA (B‐E.Coli‐16SrRNA, cat. 433291, ACD bio) following the manufacture's protocol without modifications. The distribution of positive *E. coli* 16SrRNA signal in bladder wall biopsies was assessed based on cellular morphology using light microscopy.

### 2.5 Statistical analyses

Descriptive statistics were used to describe demographic variables, clinical findings as well as histopathology, IHC, and ISH results.

### 2.6 Ethical approval and owner consent

This study involved use of surplus paraffin embedded biopsy samples, which was not subjected to ethical review. At the Faculty of Veterinary Medicine, University of Helsinki, the Pathology and Parasitology Unit holds all further rights to histopathology specimens submitted to analysis. This faculty policy is informed to clients in written. The use of client owned cadavers donated for teaching and research purposes (acquisition of control biopsies in this study) has been approved by the Viikki Campus Research Ethics Committee of the University of Helsinki, Finland (Statement 17/2021).

## 3 RESULTS

### 3.1 Study cohort

During the study period 160 cystoscopies and 106 surgical cystotomies were performed. From all, 10 dogs diagnosed with follicular cystitis were identified from the records. Two dogs were excluded, 1 because of low quality of the biopsy material and another because of insufficient biopsy material available in pathology archives. Hence, a total of 8 dogs with follicular cystitis were included in the study. All dogs were female (4/8 intact, 4/8 spayed), median age of the dogs was 2.7 years (interquartile range [IQR] 0.6‐4.9 years) and median weight of the dogs was 24.9 kg (IQR 18.8‐35.4 kg). The group comprised of 2 boxers and 1 of each of the following breeds: Bernese Mountain dog, German Shepherd dog, Labrador retriever, mixed breed, Leonberger, and Welsh Corgi Pembroke.

All dogs had a history of recurrent UTIs: median duration of clinical signs at the time of presentation was 7 months (IQR 3‐17 months) and all dogs had experienced more than 1 previous bacterial UTIs (median 4, IQR 4‐6).

The control dogs comprised of 1 Tibetan spaniel and 1 German shepherd dog. Both control dogs were euthanized for reasons other than urinary tract disease.

### 3.2 Clinical findings

**FIGURE 1:**
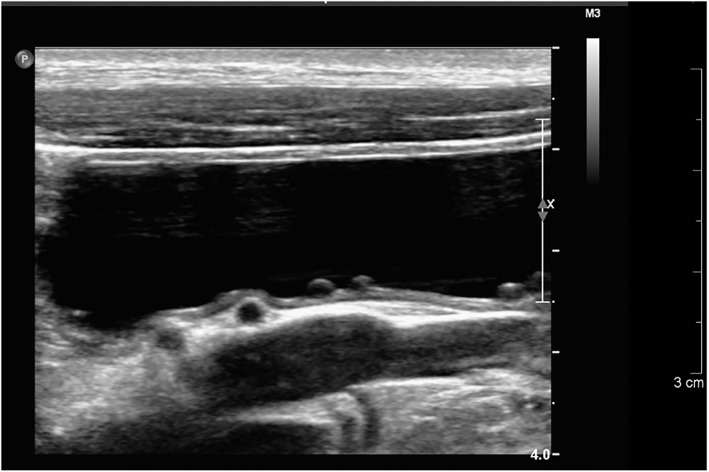
Ultrasonographic appearance of follicular bladder wall lesions in a 9 months old female boxer with a history of 6 previous bacterial urinary tract infections.

Abdominal ultrasound examination was performed in all dogs with follicular cystitis and abnormal changes in the urinary tract were observed in 5/8 dogs (irregular bladder wall n = 4, changes indicative of renal dysplasia n = 2, bladder wall polyp n = 1, ectopic enlarged ureter and hydronephrosis n = 1). Macroscopic follicles in bladder mucosa were clearly visible in ultrasound examination in 2/8 dogs (Figure 1).

**FIGURE 2:**
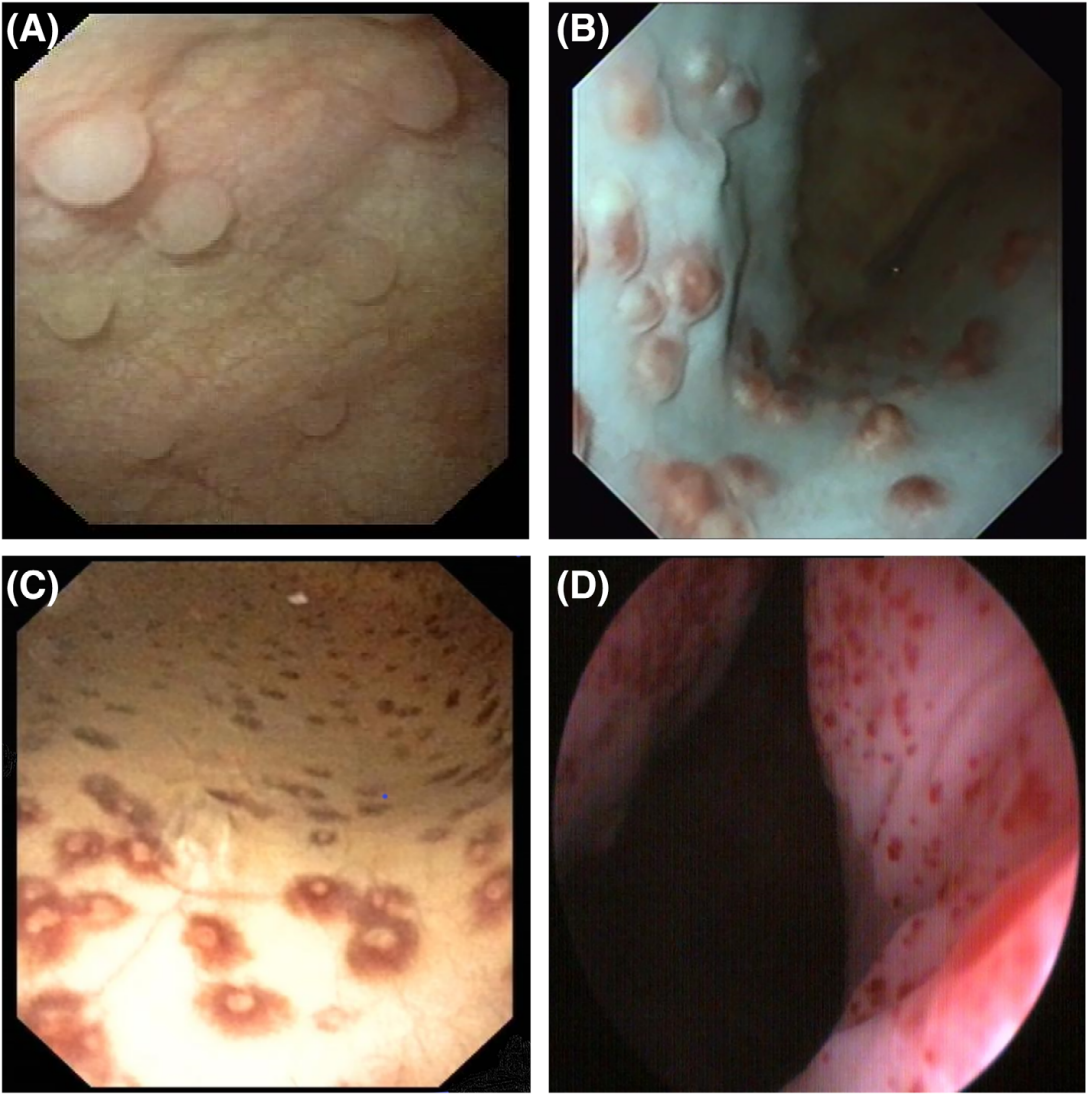
Cystoscopic appearance of follicular lesions in the bladder wall in (A) a 4‐year old female Bernese mountain dog with a history of 5 previous bacterial urinary tract infections (UTIs), (B) a 9 months old female boxer with a history of 6 previous bacterial UTIs, (C) a 6 months old female Leonberger with a history of 5 bacterial UTIs, and in (D) a 5‐year old female mixed breed dog with a history of 6 previous bacterial UTIs.

Cystoscopy was performed in 7/8 dogs and visible follicular lesions were documented in the vagina (2/7), urethra (2/7), and in the bladder wall (7/7; Figure 2A‐D). In 1 dog, bladder wall changes had a less follicular appearance and presented like petechial hemorrhages (Figure 2D). In 1 dog, follicular changes in bladder wall were visualized during surgical cystotomy for the removal of a benign bladder wall polyp and information on possible follicles in the urethra or vagina was lacking.

Possible predisposing conditions for recurrent UTIs were identified in 4/8 dogs (congfenital renal dysplasia n = 2, ectopic ureter n = 1, and hooded vulva n = 1). Additionally, 1 dog was diagnosed with a benign bladder wall polyp. At the time of examinations 3/8 dogs were receiving per oral antibiotics (amoxycillin‐clavulanic acid in all dogs) and 1 dog had received 1 intravenous dose of pre‐operative antibiotic (trimethoprim‐sulfadoxine).

### 3.3 Microbiology results

Urine culture results were available for 6/8 dogs with follicular cystitis. 2/8 samples were obtained at the time of biopsy acquisition (1 via cystocenthesis, 1 via working channel of the endoscope, no prior antimicrobials in either of the dogs), yielding pure growth of *E. coli* > 10^5^ cfu/mL in 1 dog and no bacterial growth in the other dog. 4/6 urinary samples were obtained via cystocenthesis before endoscopy (range, 3‐44 days) and bacterial growth was detected in 3/4 samples (*E. coli* > 10^5^ cfu/mL in 2/3 and *Staphylococcus pseudintermedius* > 10^5^ cfu/mL in 1/3). One of these dogs was receiving antimicrobials at the time of sampling (per‐oral amoxicillin‐clavulanic acid).

Bladder wall biopsies obtained via cystoscopy were cultured in 6/8 dogs at the time of examinations and the following bacteria were detected: *E. coli* in 5/6 (2/6 with scarce growth and 3/6 with growth only from enrichment broth) and coagulase negative *Staphylococcus* spp. (1/6, scarce growth). 3/6 dogs were receiving antibiotics at the time of endoscopy (per‐oral amoxicillin‐clavulanic acid in all cases). Detailed demographic, clinical and microbiology results are presented as Supplementary Information S1.

Urinary samples from both control dogs were negative in bacterial culture.

### 3.4 Histopathology, IHC, and ISH

**FIGURE 3:**
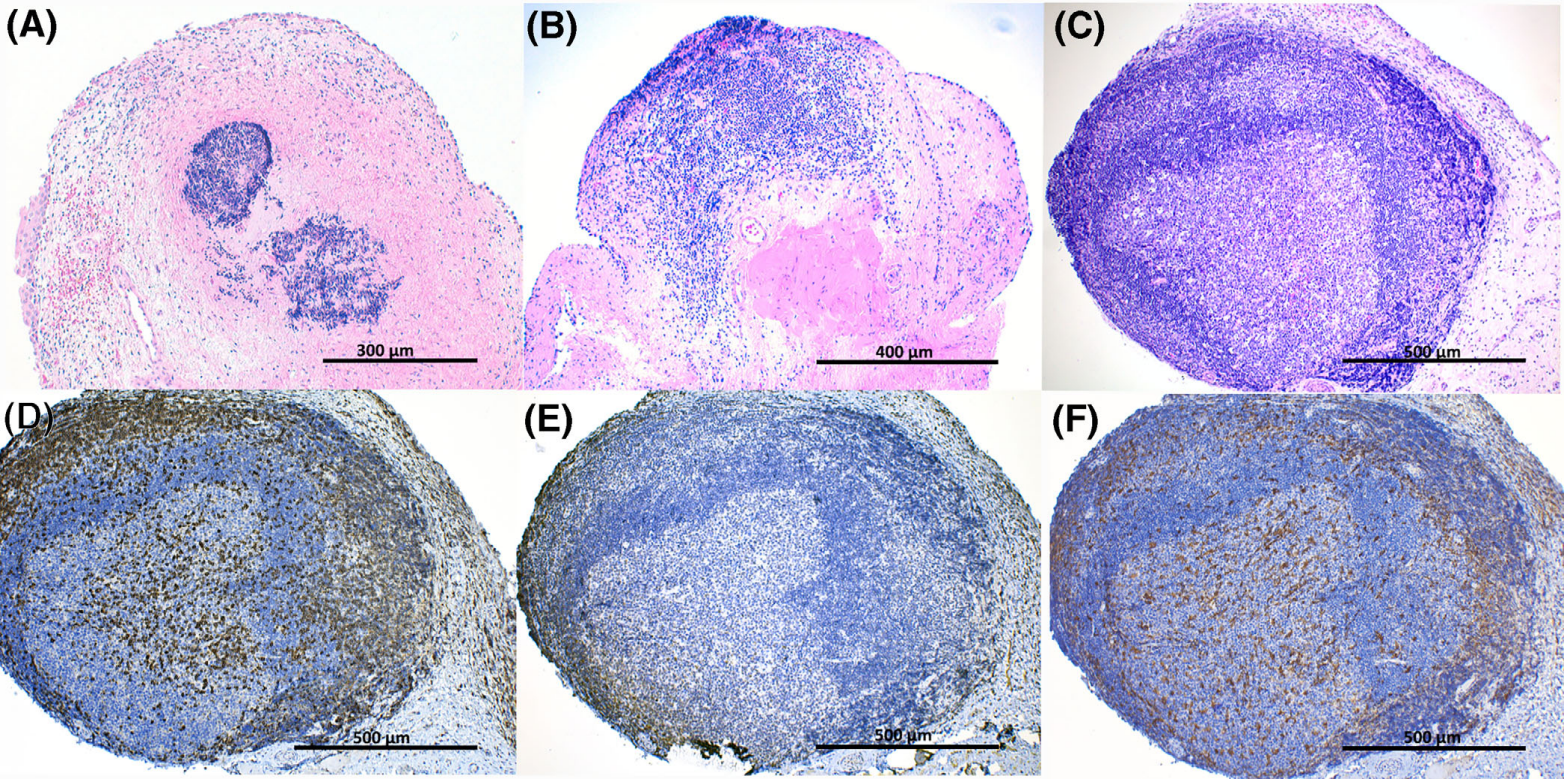
Histopathologic and immunohistochemical characterization of tertiary lymphoid structures (TLSs) in the urinary bladder of dogs diagnosed with follicular cystitis. (A) Type 1/developing TLSs composed of no more than 200 mononuclear cells. Hematoxylin and eosin stain (HE), 4×. (B) Type 2/immature TLSs formed by more than 200 mononuclear cells without specific architectural arrangement. HE stain, 4×. (C) Type 3/mature TLSs characterized by well‐defined germinal center surrounded by a mantle‐like zone and a most outer layer resembling a marginal zone. HE stain, 4×. (D‐F) Immunohistochemical characterization of the type 3 TLSs depicted in (C). CD3‐positive T‐lymphocytes are abundant in the marginal‐like zone with less numbers within the germinal center and mantle‐like zone (D). CD79α‐positive B‐lymphocytes are less in numbers and placed in the germinal center and mantle‐like zone (E). Large numbers of Iba‐1‐positive macrophages/FDCs can be seen within the germinal center and marginal‐like zone with less numbers in the mantle‐like zone (F). D‐F 4×. Higher magnifications (×20) from pictures A‐F are provided as Supplementary Material S2.

Histologically in all 8 dogs, non‐encapsulated aggregation of mononuclear cells were observed expanding the submucosa of the urinary bladder wall. Two out of 8 samples had type 1 (developing) TLSs, 3/8 showed type 2 (immature) TLSs, and 3/8 type 3 (mature) TLSs (Figure 3A‐C). Description of the macroscopic appearance of follicular cystitis and the type of TLSs observed histologically in each case is provided as Supplementary Information S1.

Type 1 and type 2 TLSs were composed of small CD3‐positive T‐lymphocytes admixed with less numbers of CD79α‐positive B‐lymphocytes and Iba‐1‐positive macrophages/FDCs without specific architectural arrangement. In 2 cases, 1 type 1 TLSs and 1 type 2 TLSs, hemorrhage was observed surrounding the nodular aggregations. Type 3 TLFs were characterized by germinal centers composed of lymphocytes displaying small nuclei and abundant pale cytoplasm (centrocytic‐type), and less numbers of lymphocytes with larger nuclei and minimal cytoplasm (centroblastic‐type). The germinal centers were surrounded by variably thick mantle‐like zones with densely packed small lymphocytes, and the most outer layer consisted of less densely packed, small and dark lymphocytes resembling a marginal zone. CD3‐positive T‐lymphocytes were abundant in the marginal‐like zones with less numbers within germinal centers and mantle‐like zones. Germinal centers and mantle‐like zones had CD79α‐positive B‐lymphocytes. Iba‐1 immunostaining showed the presence of large numbers of macrophages/FDCs within germinal centers and marginal‐like zones with less numbers of positive cells in mantle‐like zones (Figure 3D‐F).

In all evaluated biopsy samples from the urinary bladder, the submucosal stroma was mild and diffusely infiltrated by lymphocytes with a predominance of T lymphocytes (CD3‐positive) and much less B lymphocytes (CD79α‐positive), plasma cells, macrophages (Iba‐1 positive) and very scattered neutrophils. The overlying mucosa showed mild and multifocal urothelial cell hyperplasia and attenuation with variable exocytosis of T lymphocytes (CD3‐positive).

**FIGURE 4:**
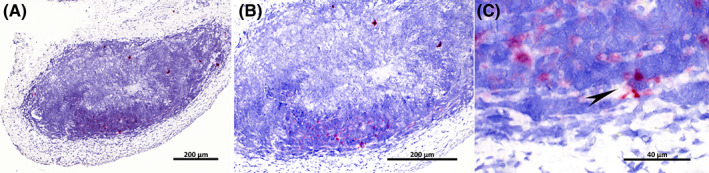
In situ hybridization specifically targeting *Escherichia coli* 16SrRNA in a type 3/mature tertiary lymphoid structures. (A) Strong red *E. coli* hybridization is present throughout the marginal‐like zone and less within the germinal center. 4×. (B) Higher magnification from (A). 10×. (C) Higher magnification from (A) and (B) depicting a cell morphologically compatible with macrophage/FDCs with intracytoplasmic *E. coli* positive signal (arrow). 60×.

**FIGURE 5:**
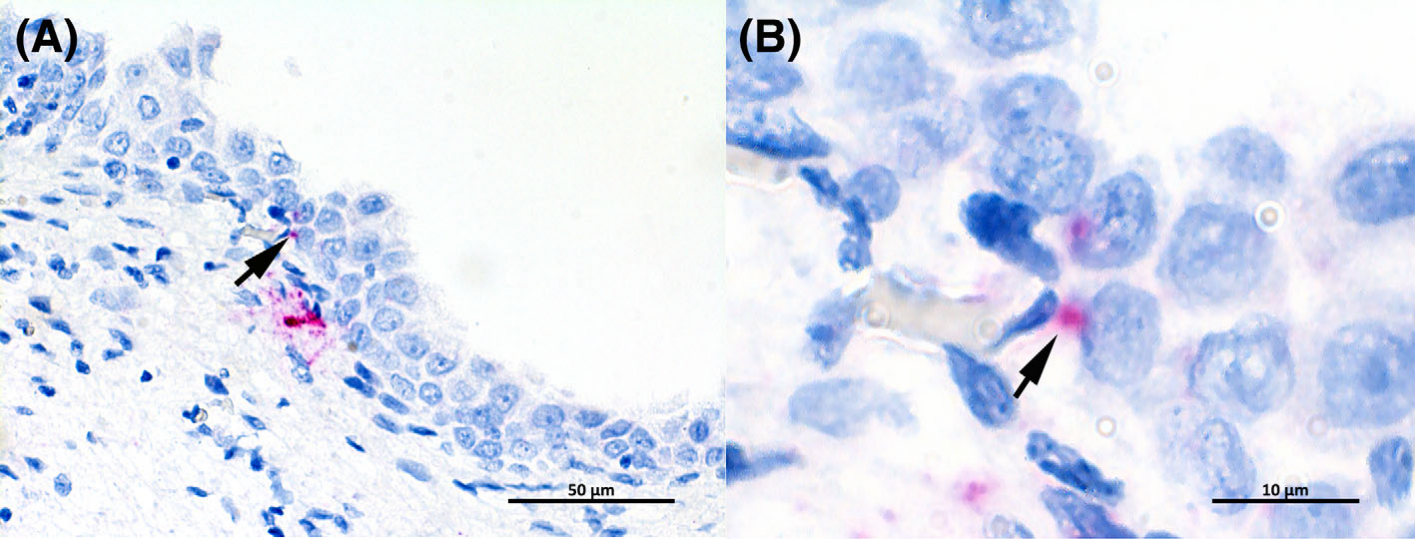
In situ hybridization specifically targeting *Escherichia coli* 16SrRNA in bladder wall biopsy from a dog with follicular cystitis. (A) Strong red *E. coli* hybridization is present within the urothelium (arrow) and in the submucosal stroma. 40×. (B) Higher magnification from A depicting urothelial cells with intracytoplasmic *E. coli* positive signal (arrow). 100×.

Positive hybridization of *E. coli* 16SrRNA by ISH was detected in all of the samples evaluated (8/8). The amount of positive hybridization in the biopsies varied from low (3/8) to moderate (4/8) and marked (1/8), though the signal was strong in all biopsies evaluated. Intrafollicular presence of *E. coli* RNA was observed in 2/2 type 1 TLSs, 2/3 type 2 TLSs and 3/3 type 3 TLSs and appeared to localize intracellular in cells morphologically compatible with macrophages/FDCs and extracellularly (Figure 4A‐C). *E. coli* hybridization was detected extracellularly within the submucosal stroma in all evaluated samples (8/8). Presence of *E. coli* RNA appearing to localize within urothelial cells, could be seen in 2/2 cases with type 1 TLSs, in 0/3 cases with type 2 TLSs, and in 1/3 with type 3 TLSs (Figure 5A‐B). Bladder wall biopsies in both control dogs did not contain inflammatory cells or TLSs, and *E. coli* ISH was negative.

## 4 DISCUSSION

The present study represents a detailed clinical and pathologic characterization of follicular cystitis in dogs linked to *E. coli* infection, and evidence that *E. coli* also localizes within developing, immature and mature/terminal TLSs besides urothelium and submucosal stroma.

In this study, follicular cystitis was diagnosed in medium and large breed female dogs and there was a strong association with chronic recurrent UTIs. Our findings are in line with observations in humans, where follicular cystitis is most often described in girls and women in connection with asymptomatic or covert UTIs.^6^, ^14^ Diagnosing recurrent UTIs in large breed female dogs is not surprising, as they are a group in which bacterial UTIs are generally more prevalent.^11^, ^21^ The predisposition of bitches to ascending UTIs is believed to results from anatomical features of the female genitourinary tract, which includes a shorter and wider urethra and a more proximal location of the urethral meatus to the anus.^21^ Additionally, other structural or developmental predisposing factors for recurrent bacterial UTIs were identified in half of the dogs included in this study.

Finding follicular cystitis in dogs with recurrent and chronic UTIs indicates that immune stimulation caused by *E. coli* infection might be an important triggering factor for the development of this condition. Mature TLSs are believed to be morphologically and functionally similar to secondary lymphoid organs such as lymph nodes and the spleen, and usually arise in a background of chronic inflammation as corroborated in this study.^22^, ^23^ Whether TLSs have a protective or noxious role by exacerbating and perpetuating an inflammatory process is however unclear.^3^, ^9^ To date, UPEC has been demonstrated within uroepithelial cells and urinary bladder stroma with high impact on antimicrobial evasion and chronicity.^24^, ^25^ We show that *E. coli* RNA is also present within cells morphologically compatible with macrophages/FDCs as early as in developing (type 1) TLSs and persist toward all maturational stages. This finding could sum in vivo evidence to recent in vitro studies demonstrating that some UPEC strains survive within mouse and human monocyte‐derived macrophages.^26^ Similar to our results, bladder wall invasion by bacteria has been described in women with recurrent UTIs and follicular lymphocyte aggregates in bladder wall biopsies.^27^

There was variable compartmentalization of *E. coli* in the urinary bladder wall, which represents a major diagnostic and therapeutic challenge.^28^ In our study, *E. coli* growth was identified in 5/6 bladder wall biopsies cultured, despite ongoing antimicrobial treatment in 3/5 dogs, but the magnitude of growth was minimal in all dogs (scarce growth or growth only after enrichment). The reduced growth of *E. coli* with routine culture methods complicates clinical decision making, as small number of bacteria might be equally associated with contamination. For bladder wall biopsies, there is currently lack of consensus on the bacterial growth threshold, which could be interpreted as clinically relevant in dogs.^29^ Our results using a combined approach of biopsy, culture and ISH can provide additional information, whether scarce bacterial growth represents a true intramural infection, since the *E. coli* probe employed in this study readily detects viable bacteria with intact 16SrRNA.^30^ ISH demonstrated *E. coli* also in 1 dog that had not received antimicrobial treatment and had negative urine and biopsy cultures, highlighting this technique as a sensitive additional diagnostic test complementing traditional mural biopsy culture in dogs with recurrent UTIs.

The ability of UPEC to invade bladder wall represents a factor, which inhibits response to certain antimicrobials.^29^ All of the dogs in our study were treated with per‐oral amoxicillin‐clavulanic acid, which is 1 of the recommended first tier antimicrobials in the treatment of UTIs in dogs.^29^ However, β‐lactam antibiotics are ineffective against UPEC in tissue, and therefore based on the common observation of bladder wall invasion in this study, the use of antimicrobials with better tissue penetration could be recommended for cases of follicular cystitis.^29^, ^31^ The ability of UPEC to form intracellular bacterial communities (IBCs) and quiescent intracellular reservoirs (QIRs) within urothelial cells constitutes another and more difficult challenge to the treatment of UPEC infections.^13^, ^32^ Intracellular environment provides UPEC a protected niche, where bacteria are virtually unaffected by host defenses and antimicrobial treatments, leading to chronic inflammation and recurrence of UTIs.^31^ In our study, positive *E. coli* signal appeared to localize within urothelial cells in 3/8 biopsies and this finding could indicate the presence of IBCs or QIRs possibly responsible for the chronic and recurrent infections seen in these dogs.

Limitations of this study include the small number of diseased dogs included as well as limited size of the control group. The retrospective nature of case identification is naturally a limitation and resulted in variable antimicrobial treatments in these dogs and inconsistent sample acquisition for urine and biopsy cultures. However, follicular cystitis is a relatively uncommon finding in dogs and therefore timely prospective case recruitment might be unrealistic at least without a multicenter study approach. Another important limitation was the small size of endoscopic urinary bladder wall biopsies, which naturally represented only a fraction of the entire bladder mucosa. Additionally this study only addressed the role of an intramural *E. coli* infection in dogs diagnosed with follicular cystitis and there is a need for future studies looking at the prevalence and role of intramural infections in other chronic lower urinary tract diseases.

The RNA‐based method of bacteria identification identifies both dormant and metabolically active live bacteria, as both produce rRNA, and the method is superior to DNA‐based methods in the identification of live bacteria.^30^ However, the 16SrRNA sequencing used in this study can also identify RNA from dead bacterial cells, which is a limitation in our study, as advanced laboratory methods were not employed to demonstrate replicative and infectious capacity of the *E. coli* identified in the biopsies.^30^ Furthermore, the cultured *E. coli* were not subject to whole genome sequencing and therefore, identification of specific strains, antimicrobial resistance gene profile, and virulence factors that could potentially indicate ability to survive and replicate within macrophages and urothelial cells was not possible.

To conclude, follicular cystitis was diagnosed in female dogs with chronic urinary bladder inflammation caused by recurrent bacterial UTIs. Intramural *E. coli* bacteria were demonstrated in bladder wall biopsies in all dogs, localizing in the urothelium, submucosal stroma and also within cells morphologically compatible with macrophages/FDCs in developmental, immature, and mature TLSs, representing a potential triggering factor for the development of follicular cystitis.

## CONFLICT OF INTEREST DECLARATION

Authors declare no conflict of interest.

## OFF‐LABEL ANTIMICROBIAL DECLARATION

Authors declare no off‐label use of antimicrobials.

## INSTITUTIONAL ANIMAL CARE AND USE COMMITTEE (IACUC) OR OTHER APPROVAL DECLARATION

This study involved use of surplus paraffin embedded biopsy samples, which was not subjected to ethical review. At the Faculty of Veterinary Medicine, University of Helsinki, the Pathology and Parasitology Unit holds all further rights to histopathology specimens submitted to analysis. This faculty policy is informed to clients in written. The use of client owned cadavers donated for teaching and research purposes (acquisition of control biopsies in this study) has been approved by the Viikki Campus Research Ethics Committee of the University of Helsinki, Finland (Statement 17/2021).

## HUMAN ETHICS APPROVAL DECLARATION

Authors declare human ethics approval was not needed for this study.

## Supporting information


**Data S1:** Supporting Information

**Data S2:** Supporting Information
