# Correction to “*Escherichia coli*‐associated follicular cystitis in dogs: Clinical and pathologic characterization”

**DOI:** 10.1111/jvim.16834

**Published:** 2023-07-27

Viitanen SJ, Tuomisto L, Salonen N, Eskola K, Kegler K. Escherichia coli‐associated follicular cystitis in dogs: Clinical and pathologic characterization. J Vet Intern Med. 2023;37(3):1059‐1066. doi:10.1111/jvim.16719

In the originally published article, the author's name Katarina Eskola was spelled incorrectly. This has been corrected in the version of record on Wiley Online Library.

The authors regret for this error.

