# Supplementary material for: Escherichia coli ‐associated follicular cystitis in dogs: Clinical and pathologic characterization
Source: J Vet Intern Med. 2023 May 8;37(3):1059–66. doi: 10.1111/jvim.16719 (PMC10229364; doi:10.1111/jvim.16719)
Supplement: Supplementary file 1 — Data S1: Supporting Information [file JVIM-37-1059-s002.pdf]

S1 Demographic and clinical characteristics of dogs diagnosed with follicular cystitis (n=8)

**No.**	**Breed**	**Sex**	**Age (years)**	**Duration of clinical signs (months)**	**Number of previous bacterial UTIs**	**Predisposing diseases identified**	**Urine culture**	**Antibiotics at biopsy acquisition**	**Bladder wall biopsy culture**	**Macro-scopic bladder wall lesions**	**Type of TLSs**	**Location of positive *E.coli* ISH**
1	Labrador retriever	FS	4,3	36	8	Renal dysplasia, ectopic ureter	*S. pseudintermedius* >10^5 cfu/ml<br>17 days prior to biopsies	AMC	Koagulase negative *Staphylococcus* spp.<br>Scarce growth	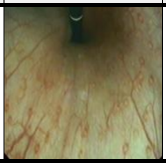	Type 2	submucosal<br>intrafollicular
2	Boxer	F	0,8	7	6	None identified	*E. coli* >10^5 cfu/ml<br>44 days prior to biopsies	AMC	*E.coli*<br>Scarce growth	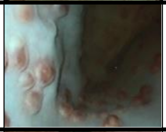	Type 3	submucosal<br>intraepithelial<br>intrafollicular
3	Boxer	F	1,1	8	5	Renal dysplasia	*E. coli* >10^5 cfu/ml<br>3 days prior to biopsies	None	*E. coli*<br>Enrichment broth	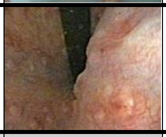	Type 2	submucosal<br>intrafollicular
4	Welsh corgi Pembroke	FS	10,3	3	2	Benign bladder wall polyp	Negative<br>12 days prior to biopsies	Single dose TMS			Type 1	submucosal<br>intraepithelial<br>intrafollicular
5	Mixed breed	FS	5	22	6	None identified		None	*E. coli*<br>Enrichment broth	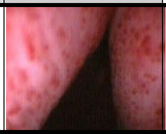	Type 2	submucosal
6	Bernese mountain dog	FS	4,5	12	5	Hooded vulva	*E. coli* >10^5 cfu/ml<br>At biopsy acquisition	None		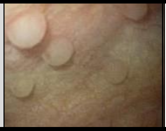	Type 3	submucosal<br>intrafollicular
7	German shepherd dog	F	0,5	2,5	2	None identified		AMC	*E. coli*<br>Enrichment broth	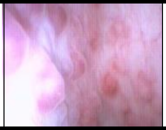	Type 3	submucosal<br>intrafollicular
8	Leon-berger	F	0,5	3	5	None identified	Negative<br>At biopsy acquisition	None	Negative	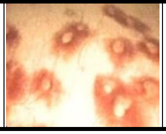	Type 1	submucosal<br>intraepithelial<br>intrafollicular

F= female, FS= female spayed, UTI=urinary tract infection, AMC=Amoxycillin-clavulanic acid, TMS= Trimethoprim-sulphadoxine, TLS=Tertiary lymphoid structure, ISH=*in situ* hybridization
