# Supplementary material for: Escherichia coli ‐associated follicular cystitis in dogs: Clinical and pathologic characterization
Source: J Vet Intern Med. 2023 May 8;37(3):1059–66. doi: 10.1111/jvim.16719 (PMC10229364; doi:10.1111/jvim.16719)
Supplement: Supplementary file 2 — Data S2: Supporting Information [file JVIM-37-1059-s001.pdf]

S2 Histopathologic and immunohistochemical characterization of tertiary lymphoid structures in the urinary bladder of dogs diagnosed with follicular cystitis. Higher magnifications (x20, Aa-Ff) from areas marked with a rectangle for Pictures 3 A-F in the manuscript. Hematoxylin and eosin stain (A-C and Aa-Cc), CD3 immunohistochemical stain (D and Dd), CD79α immunohistochemical stain (E and Ee) and Iba-1 immunohistochemical stain (F and Ff).

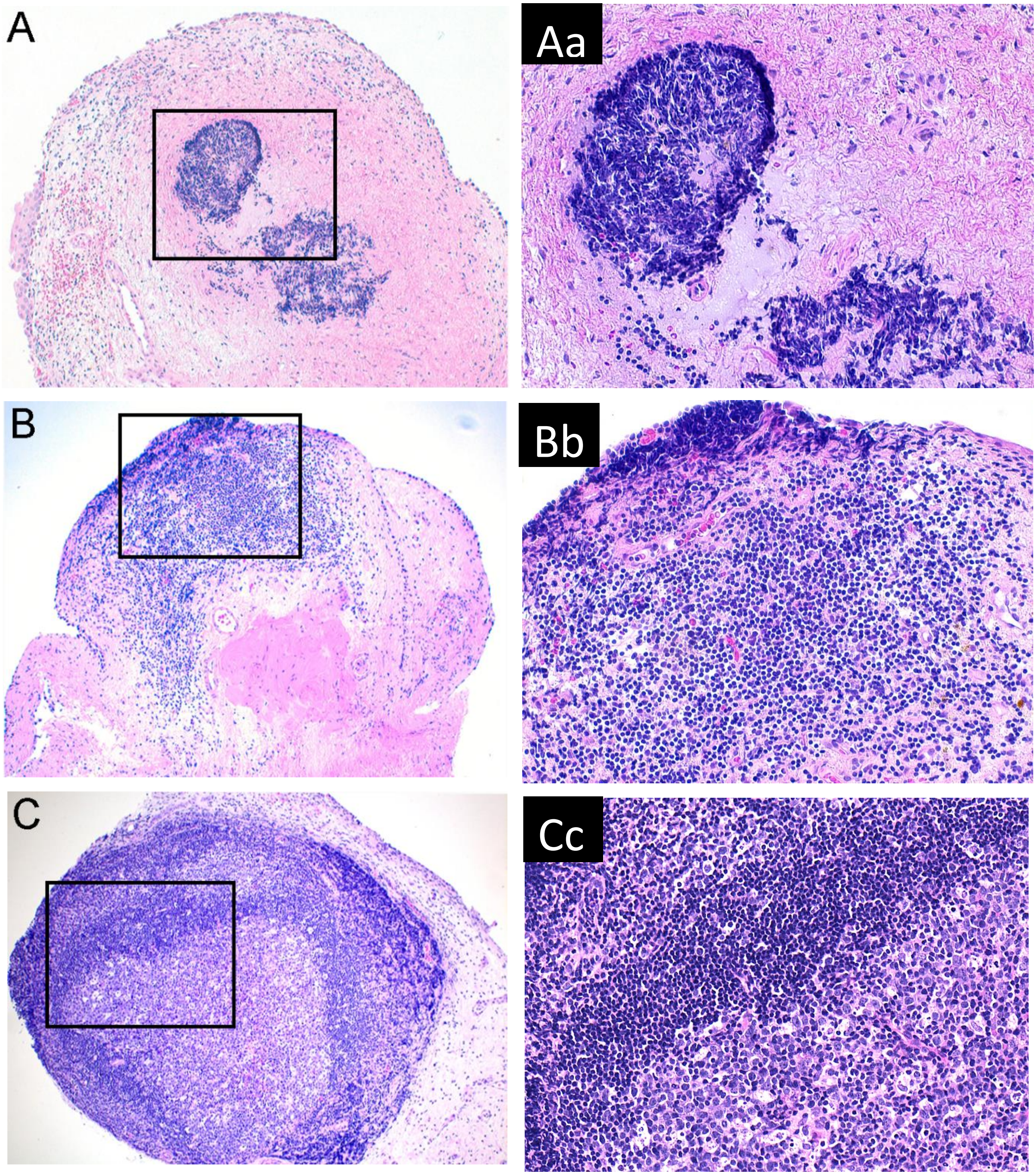

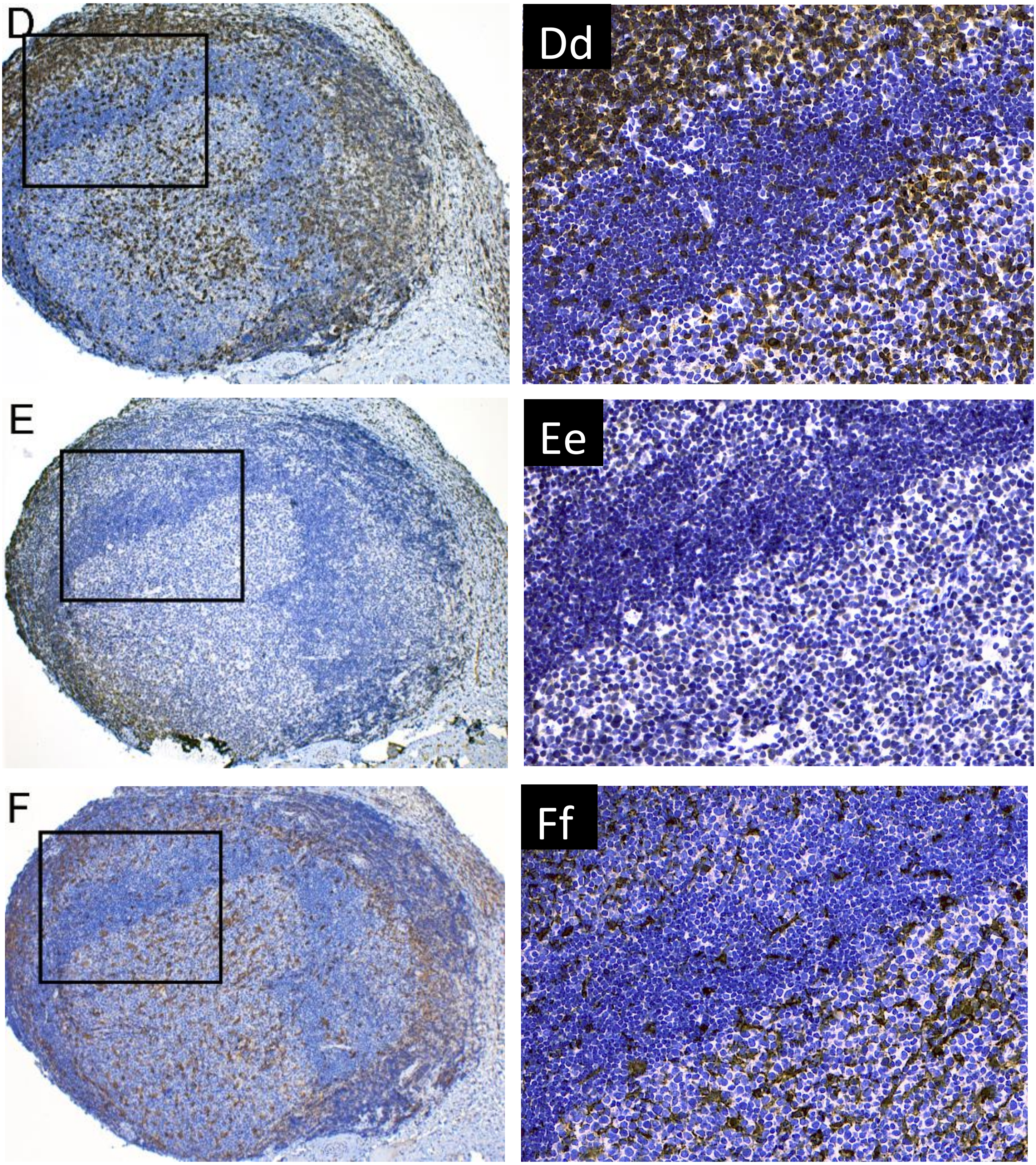
D
Dd
E
Ee
F
Ff
